# Evaluation of restricted access media for the purification of cell culture‐derived Orf viruses

**DOI:** 10.1002/elsc.202300009

**Published:** 2023-07-14

**Authors:** Keven Lothert, Yasmina M. J. Harsy, Patrick Endres, Egbert Müller, Michael W. Wolff

**Affiliations:** ^1^ Institute of Bioprocess Engineering and Pharmaceutical Technology University of Applied Sciences Mittelhessen (THM) Giessen Germany; ^2^ Tosoh Bioscience GmbH, Separations Business Unit ‐ Europe Griesheim Germany

**Keywords:** downstream processing, multimodal chromatography, parapoxvirus ovis, platform technology, viral vector

## Abstract

Recently, multimodal chromatography using restricted access media (RAM) for the purification of nanoparticles, such as viruses has regained increasing attention. These chromatography resins combine size exclusion on the particle shell and adsorptive interaction within the core. Accordingly, smaller process‐related impurities, for example, DNA and proteins, can be retained, while larger product viruses can pass unhindered. We evaluated a range of currently available RAM, differing in the shells’ pore cut‐off and the core chemistry, for the purification of a cell culture‐derived clarified model virus, namely the Orf virus (ORFV). We examined impurity depletion and product recovery as relevant criteria for the evaluation of column performance, as well as scale‐up robustness and regeneration potential for evaluating a multiple use application. The results indicate that some columns, for example, the Capto Core, enable both a high DNA and protein removal, while others, for example, the Monomix Core 60 (MC60), are more suitable for DNA depletion. Furthermore, column regeneration is facilitated by using columns with larger shell pores (5000 vs. 700 kDa) and weaker binding interactions (anion exchange vs. multimodal). According to these findings, the choice of RAM resins should be selected according to the respective feed sample composition and the planned number of application cycles.

**Abbreviations**
CC (700 / 400): Capto Core (size cut‐off 700 / 400 KDa)
CV: column volume
DSP: downstream processing
MC60: Monomix Core 60
ORFV: Orf virus
RAM: restricted access media
USP: upstream processing
VLP: virus‐like particle
VP: ViralPolish

## 1 INTRODUCTION

Several medical challenges are currently targeted by using viruses or virus‐like particles (VLPs), which can be classified as biological nanoplexes. These include, among others, classical vaccination approaches with (live) attenuated viruses [1], VLPs, as well as viral vectors for gene and tumor therapy [2, 3]. The amplification of sufficient amounts of the product virus is typically achieved during upstream processing (USP), using susceptible host cells [4], however, USP is only one part of the entire production process. For a safe and efficient application in humans, product purity requirements by regulatory authorities must be met [5]. As the cell culture harvest contains a variety of process‐related impurities, such as cell debris, host cell proteins, and DNA, a downstream process (DSP) for the purification of the product, following its cell‐based amplification, is mandatory [6].

Due to a complex impurity composition, the DSP of cell culture‐derived viruses usually involves several steps, employing a combination of various techniques, such as chromatography, extraction, filtration, and centrifugation procedures [7]. Although chromatography often involves a sophisticated method optimization [8], it remains the backbone of many virus DSPs [6]. Versatile chromatography techniques are available, such as ion exchange, hydrophobic interaction, or size exclusion, and can be selected according to respective product properties. Most of these techniques are adsorption methods, which require a rigorous optimization to enable a separation of product and impurities [6, 9]. Moreover, product losses can occur due to strong adsorption, as described, for example, for parapoxviruses [10]. Although an application in a product flow‐through mode is possible, as reported for influenza viruses [11, 12], running conditions must be adjusted individually for each new product. Another classical flow‐through technique, the size exclusion chromatography, is characterized by a low productivity, as only a fraction of the column volume (CV) (∼15%) can be loaded as a sample [11, 13].

To reduce the challenges for optimization, for scale‐up, as well as for a process transfer to a production facility, scalable modular platform approaches are preferable. These are particularly beneficial in the case of different virus products or different genotypes of the same virus, as viral vector platforms allow for a modification of the virus composition, for example, the expression of certain (structural) proteins [14].

**PRACTICAL APPLICATION**

Chromatographic RAM provide a scalable possibility to gently process virus particles, while successfully removing major contaminants such as host cell DNA and proteins. We compared different RAM resins with a varying shell pore cut‐off and core ligand binding chemistry for the purification of cell culture‐derived Orf virus particles. From this, conclusions can be drawn for future applications in the field of virus purification with respect to the product and the applied manufacturing process. Furthermore, we show the reproducibility of the virus recovery and the impurity depletion during single‐use and after repeated cleaning, and compared two different process scales. This allows other users to select the appropriate column matrix with regard to their application and process requirements.

One potential solution to overcome the above‐mentioned challenges of the referred classical chromatography methods is the purification of viruses by mixed‐mode resins using restricted access media (RAM) [15]. Generally, RAM resins combine size exclusion effects of the shell with adsorptive interactions in the core [16]. The shell pores do not allow a passage of larger particles, such as viruses, thus excluding them from entering the core bead. As a result, a gentle processing of the viral product is enabled, as the smaller impurities can enter the ligand‐activated core and are removed from the product stream [17]. As this separation principle is based on a product flow‐through mode, it is usually applied at a later processing stage using smaller feed volumes for the removal of residual contaminants [18, 19, 20, 21, 22], but it is also described in literature for the main (primary) purification step [23]. Product recoveries of 85%–100% for various viruses indicate the platform character of the RAM purification approach. In combination with a high product purity and short processing times, it shows the superior performance of RAM materials over classical size exclusion chromatography. Moreover, RAM also enables lower product losses than classical bind‐and‐elute methods using comparable anion exchange ligands alone, for example, for influenza [21, 24].

In all currently published RAM applications, Capto Core (CC), the first commercially available RAM resin was used. CC is based on cross‐linked agarose beads with the core ligands consisting of octylamine [15, 25]. The latter is both hydrophobic and positively charged, allowing a multifaceted adsorption of impurities. Recently, several other RAM products entered the market, offering different specifications with regard to the shell pore size and the type of the core ligand. Although there are no publications describing the use of these novel materials for virus purification, the availability of RAM columns with varying core and shell structures allows for a more targeted usage of the RAM technology, depending on the individual composition of the feed material and the position within the DSP train.

In a recent study, the CC700 performance was compared to other commonly applied chromatographic methods in terms of recovery, impurity depletion [10], and with regard to the positioning of the method in the DSP for the purification of the Orf virus (ORFV) [18]. Due to the promising performance of CC700 in this process and the current interest in the ORFV as a vector platform for various pharmaceutical applications [26], the ORFV was selected as a model virus to compare various RAM technologies. ORFVs have a brick‐ or oval‐shaped morphology, and their size ranges in length from 220–450 nm, and in width and depth from 140–260 nm [18, 26, 27].

As RAM resins promise a straightforward translation to the required process scale and a suitability for a broad range of different viruses, we evaluated to what extent a purification of the model ORFV is affected by variations in the RAM ligand and the shell composition. For this purpose, currently, commercially available RAM resins were compared for an application as a purification step following clarification. All resins had an inert polysaccharide‐based particle shell and varied in the shells’ pore size (400 to 5000 kDa), the binding chemistry of the core (cation‐, anion exchange, multimodal) and the column size (due to the availability of columns). The performance of the purification procedure was characterized with regard to product recovery and impurity depletion as well as the individual columns’ capacity during sample loading. Furthermore, the repeated utilization with intermediate cleaning was examined, in order to evaluate whether the columns are more suitable for multiple‐use applications, or whether a single‐use application might be recommended. This is of particular interest, if continuous chromatography methods are to be used.

## 2 MATERIALS AND METHODS

### 2.1 Model virus production and clarification

The ORFV, which expresses a green fluorescent protein in infected cells, was selected as a model virus and propagated in an adherent cell culture, as previously described [10]. Briefly, Vero cells (American Type Culture Collection) were seeded at 2E+04 cells per cm^2^ and infected with a multiplicity of infection of 0.05. Five days after infection, the cells were disrupted by one freeze/thaw cycle (−80°C, 22°C) and the supernatant was collected.

The virus harvest was then clarified, using a serial two‐step depth filtration with decreasing pore sizes (5 and 0.65 μm). Polypropylene filters (Sartopure PP3, Sartorius Stedim Biotech GmbH) were applied and the filtration process was carried out at a constant feed pressure of 0.05 MPa, using the Sartoflow Smart system operated by the Biopat MFCS 4 software (Sartorius Stedim Biotech GmbH). Harvests of different culture flasks were pooled, aliquoted, and stored at −80°C after clarification. For all subsequent experiments, the same clarified feed suspension was used.

### 2.2 Chromatography

An Äkta Pure 25 system (Cytiva), operated by the Unicorn 7.7 software, with online monitoring of UV (280 nm), conductivity, and light scattering (Nano DLS Particle Size Analyzer, Brookhaven Instruments), was used for the chromatographic runs. Before usage, all buffers were filtered through a 0.2 μm bottle top filter (Corning) and degassed by ultrasonication (USC500 THD, VWR).

Small‐scale (1 mL) RAM columns were obtained from Cytiva (Capto Core) and BioToolomics (SepFast DUO Q/S, ViralPolish (VP) A/B) with the specifications indicated in Table 1. The CC700 was considered as the reference material for all evaluations, as it was the first commercially available RAM material with several literature reports. The same chromatography protocol was applied for all columns, using a constant flow rate of about 1 CV min^−1^ (1 mL min^−1^). The procedure comprised the following six steps: (i) equilibration, using 20 mM TRIS supplemented with 180 mM NaCl at a pH of 7.4 for 5 CV, (ii) loading of the clarified ORFV harvest, (iii) washing, with the equilibration buffer for 5 CV, (iv) elution of the bound impurities, with 7 CV of 20 mM TRIS supplemented with 2 M NaCl, pH 7.4, (v) cleaning, with 5 CV of 1 M NaOH, and (vi) washing, with 6 CV water. The loading (ii) was done until an increase in the protein breakthrough of 10%–20% of the feed material was detected by UV_280_ monitoring. This resulted in different load volumes, ranging between 8 and 20 mL, depending on the column type (see Table 1). For some columns, no protein retention under the evaluated conditions could be achieved, as the UV signal exceeded the stop criterion immediately after the void volume passed through the column, thus allowing no detection of an additional increase. Therefore, these columns were loaded with a fixed volume of 8 CV, as a comparison to the lowest possible loading volume of other columns with a protein retention.

The performance of the CC400 and CC700 columns was evaluated in triplicates, using a new column for each run to estimate the error of replicates. As, accordingly, the batch deviation of product recovery and impurity depletion for fresh columns was negligible, subsequently, all other columns were analyzed with single runs, using fresh columns only.

Furthermore, the regenerability of selected columns (CC400, CC700, and all columns with a cut‐off of 5000 kDa) was evaluated by re‐using the same column three times after NaOH (1 M) and H_2_O cleaning.

Ultimately, the small‐scale experiments were supplemented for evaluations on a larger scale, using 4.7 mL CC700 (Cytiva) and 4.2 mL Monomix Core 60 (MC60, Sepax Technologies, Inc.) columns. The chromatographic procedure was equal to the smaller scale with regard to the number of CV of buffer and feed in the individual steps. The flow rate was adjusted volumetrically to equal 1 CV min^−1^ or was set to the maximum possible flow rate, resulting in 3.7 mL min^−1^ for the CC700, corresponding to 477 cm h^−1^, the maximum for that column, and 4.2 mL min^−1^ for the MC60, corresponding to 570 cm h^−1^, half of the maximum. To support the online monitoring, the flow‐through was fractionated, and samples were taken for each loaded CV.

### 2.3 Analytics

The chromatographic fractions (clarified feed, flow‐through, wash, and elution) of all runs were analyzed for the contained amount of the infective viruses, total protein, and DNA. The protein and DNA levels were determined using the Pierce BCA Protein Assay and the Quant‐iT PicoGreen dsDNA Assay Kits (both Thermo Fisher Scientific) according to the manufacturers’ instructions, as previously described [10].

The quantification of infective virus particles was done by an automated flow cytometry‐based approach, as previously described [18]. Briefly, Vero cells were infected with a standard virus stock, ranging between 1.5E+05 and 1.0E+07 infective units (IU) mL^−1^, the medium blank, or the respective chromatography sample. After 16 h of incubation, the percentage of fluorescent cells, due to the GFP expression after virus infection, was determined, using a flow cytometer (Guava easy Cyte HT). The infective virus concentration of the chromatographic samples was estimated by a linear calibration curve, based on an in‐house standard. The general standard deviation of the assay for duplicate measurements was less than 10%.

## 3 RESULTS AND DISCUSSION

### 3.1 RAM column performance on first use

**FIGURE 1:**
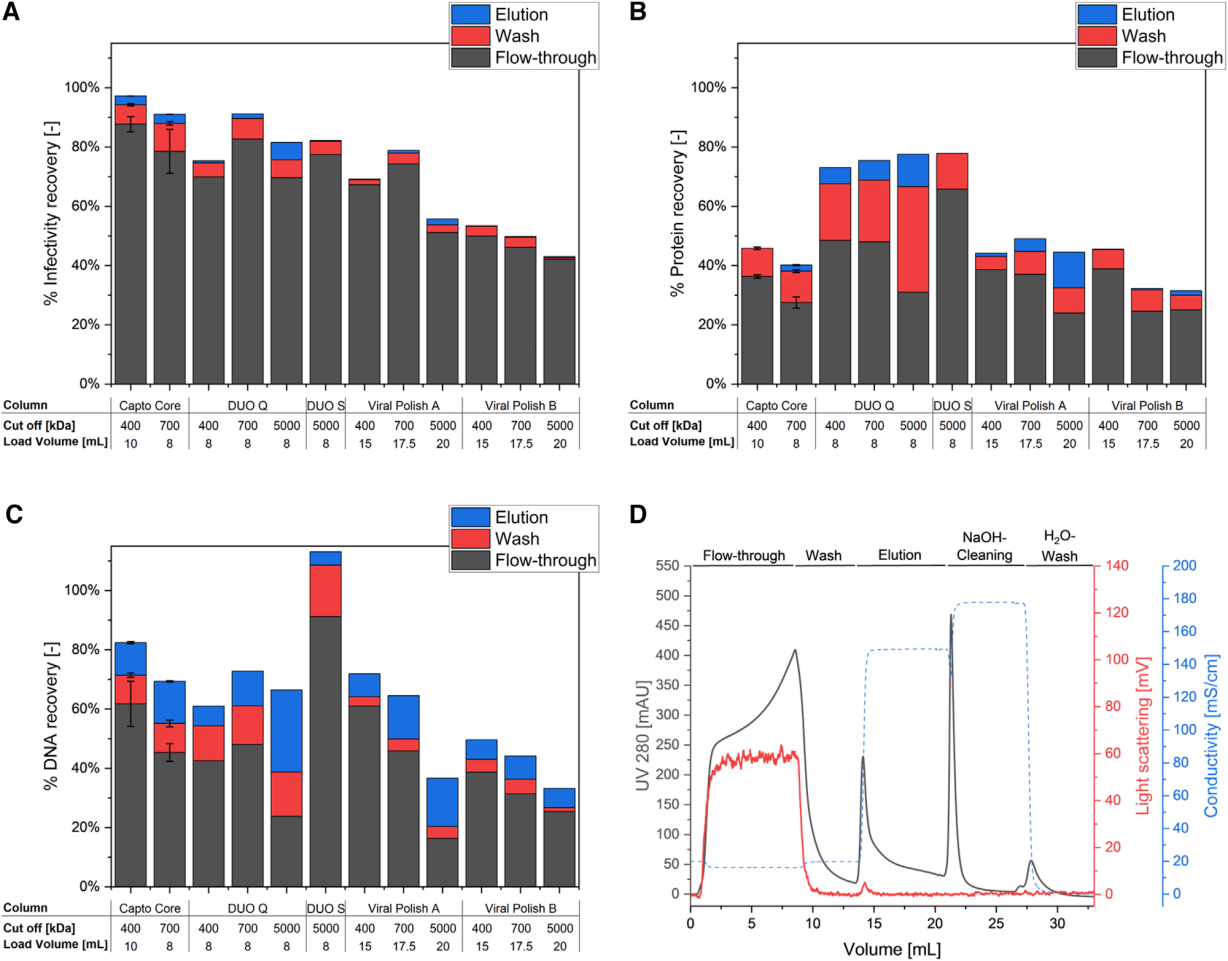
Orf virus purification performance of different restricted access materials (RAM) on single‐use application of 1 mL columns. Depicted are the recovery of (A) infective viruses, (B) total protein, and (C) total DNA amounts in the individual chromatographic fractions of flow‐through, wash, and elution. The values in percent are in relation to the amount contained in the feed material. Error bars for the first two columns display the standard deviation of triplicate chromatographic runs. All other runs were performed once, as a proof of concept. An example chromatogram (D) is shown for the Capto Core 700 (CC700) purification, showing UV_280_ (black), light scattering (red), and conductivity monitoring (dashed) during the different phases of purification. For all columns, sample loading was stopped after the protein breakthrough increased above 10%–20%, based on UV detection (hereafter 8 mL load).

At first, twelve different 1 mL RAM columns were assessed (Table 1) for the purification of the model virus, ORFV. All columns (single‐use) were loaded until the UV signal indicated a protein breakthrough of 10%–20%. The reference for all runs was the CC700 column, as it is the current standard material for comparable RAM applications during virus purification. CC700 allowed a loading of about 8 mL feed suspension until an exponential increase of the UV signal started (Figure 1D). Under the same process conditions, the DUO Q columns could also be loaded with 8 mL feed before reaching the stop criterion. In contrast, the DUO S did not enable a considerable retention of proteins, based on UV detection, and was accordingly loaded with 8 CV (8 mL) to allow a comparison with the CC700 reference. The highest amount of feed could be loaded onto the VP A and B columns with loading volumes of 15, 17.5, and 20 mL for shell cut‐offs of 400, 700, and 5000 kDa, respectively (Table 1). However, the increased loading capacities of the VP columns were paralleled by a reduced recovery of the infective viruses (Figure 1A). Although the majority of the recovered viruses for all tested columns was found in the flow‐through fraction, the amount was the highest for CC, DUO Q and S, and the VP A (700 kDa shell cut‐off) with values ranging between 70%–87% (Table 1, Figure 1A). According to the standard deviation of the assay, and the deviation of technical triplicate runs estimated by CC400 and CC700, these results can be considered as comparable. Furthermore, the data is in good accordance with current literature, presenting virus and VLP recovery yields for CC700 purifications of 85%–100% [11, 18, 22, 23]. On the contrary, VP A (5000 kDa cut‐off) and VP B (all cut‐off sizes) resulted in the lowest product recoveries of 42%–51% in the flow‐through.

**TABLE 1:** Evaluated RAM columns that were tested on a 1 mL CV scale with the respective properties of the individual materials. Shown are the total amounts of infective virus, protein, and DNA that were loaded and subsequently recovered in the individual chromatographic fractions.

Column	Capto Core		Sep Fast DUO Q			Sep Fast DUO S	Viral Polish A			Viral Polish B		
**Shell cut‐off [kDa]**	400	700	400	700	5000	5000	400	700	5000	400	700	5000

Matrix material	Cross‐linked agarose		Cross‐linked polysaccharide composite beads				Agarose beads			Agarose beads			
Core material (interaction principle)	Octylamine (anion exchange + hydrophobic interaction)		Quaternary (anion exchange)			Sulfopropyl (cation exchange)	Mixed mode (anion exchange + mild hydrophobic interaction)			Mixed mode (anion exchange + strong hydrophobic interaction)			
**Load volume [mL] onto 1 mL column**	10	8	8	8	8	8	15	17.5	20	15	17.5	20	
**Infective virus recovery [IU]**	Feed	4.5E+07	3.4E+07	4.4E+07	4.4E+07	4.4E+07	4.4E+07	8.3+E07	9.7+E07	1.1+E08	8.3+E07	9.7+E07	1.1+E08
	Flow‐through	3.9E+07	2.7E+07	3.1E+07	3.7E+07	3.1E+07	3.4E+07	5.6E+07	7.2E+07	5.6E+07	4.2E+07	4.6E+07	4.6E+07
	Wash	2.9E+06	3.2E+06	1.3E+06	2.0 E+06	1.7E+06	1.2E+06	4.6E+05	1.0E+06	7.0E+05	9.00E+05	9.5E+05	1.4E+05
	Elution	1.3E+06	1.0E+06	2.9E+05	6.1E+05	2.3E+06	1.5E+05	9.1E+04	3.6E+05	7.7E+05	8.4E+04	1.1E+05	2.3E+05
**Total protein recovery [μg]**	Feed	23,000	18,000	20,000	20,000	20,000	20,000	40,000	46,000	53,000	40,000	46,000	53,000
	Flow‐through	8400	4800	9600	9510	6140	13,020	15,310	17,160	12,700	15,440	11,380	13,240
	Wash	2200	1900	3780	4120	7060	2390	1780	3540	4480	2560	3320	2620
	Elution	<20	360	1090	1310	2160	<20	450	1990	6370	47	255	800
**Total DNA recovery [ng]**	Feed	4300	3900	4600	4600	4600	4600	13,000	15,000	17,000	13,000	15,000	17,000
	Flow‐through	2700	1800	1980	2230	1100	4230	7990	7010	2860	5070	4800	4430
	Wash	420	380	544	603	694	806	415	616	697	571	753	228
	Elution	480	550	309	544	1280	210	1010	2230	2840	853	1190	1140

*Note*: Total values for Capto Core depict mean of technical triplicates.

The wash and elution fractions of all columns contained minor virus amounts with less than 5%–9% (Table 1, Figure 1D). Accordingly, viruses that were not recovered, either remained on the column, or were inactivated. As the data suggests a correlation of the product losses with the pore size of the shell and the binding mechanism of the ligand, for example, VP B has a stronger hydrophobic interaction than A, it can be assumed that the virus has access to the inner core through the large pores and, thus remains on the column. For the DUO S and Q, these effects were not observed, even at a 5000 kDa cut‐off. Hence, the ancillary hydrophobic interaction of the VP columns caused the additional product losses. However, irrespective of the ligands within the particle core, the ORFV (∼200 nm) is unlikely to enter the shell pores to bind to these ligands [28]. Nevertheless, the complex surface composition of viruses could enable the partial intrusion of viral surface proteins into the pores, thus leading to the retention of the whole virus particle. Furthermore, viruses could be retained by adhering to longer host cell DNA strands, bound to the core and protruding from the pores [29], due to irregularities in the inert shell, exposing core structures on the surface, or due to an unspecific binding to the outer shell. This unspecific retention of the virus product was observed for all evaluated columns, leading to open material balances to varying degrees, as has occasionally been described for an anion exchange purification of viruses. In addition to an unwanted product retention, an inactivation of the product could also occur. Depending on the composition of the particle resin and the packing of the column, the shear forces can vary. A possible inactivation could be evaluated by using different, for example, lower, flow rates or an orthogonal quantification approach without detecting infectivity. However, detailed evaluations would have been beyond the scope of this study, which is aimed at a rapid purification process to obtain an infectious virus product.

Considering the impurity removal, the highest protein amounts of 48%–65% in the product fraction were observed with DUO Q 400, 700, and DUO S 5000 columns (Table 1, Figure 1B). The lowest protein amounts in the fraction were determined for the CC700, VP A 5000, VP B 700, and 5000, with about 24%–27%. As the loading was stopped at an UV_280_ signal indicating an exponential increase of about 10%–20% of the maximum signal (i.e., feed material in bypass), up to 20% of the proteins were to be expected in the breakthrough. Accordingly, this value could be further reduced, by stopping the loading before a more pronounced breakthrough occurs. This, in turn, would impair the process productivity, as less feed material can be handled while the column capacity is not fully exploited. In return, the obtained data suggests a constant protein breakthrough of 4%–7% from the start of the loading procedure for the best performing columns. In the case of the cationic DUO S column, it is unlikely to achieve an improvement at neutral pH conditions, requiring an alteration of the pH value of the sample [10]. As many viruses are stable in a narrow pH range, pH variations are not suitable for the majority of viruses [7, 9, 30]. For the mixed‐mode or anion exchange ligands, an improvement in the protein retention could be enabled by a dilution or a dialysis of the feed, thus reducing the loading buffers’ conductivity, or by lowering the flow rate, thus increasing the residence time. This is supported by the observation that a higher protein depletion was achieved by an increasing shell cut‐off for all tested columns (Figure 1B). The larger pores in the shell facilitate a higher diffusion rate of the contaminating protein components into the particle, which was also previously observed for proteins and latex nanoparticles, when comparing CC700 and CC400 [28]. In this study, residence times of at least 2 min were suggested for loading a feed containing 1 mg_protein_ mL^−1^, whereas in the present experiments, residence times of 1 min were applied, using feed containing more than 2 mg mL^−1^ contaminating proteins in order to reduce the process time.

The DNA recovery in the flow‐through (product fraction) was between 17% and 92% (Figure 1C). As for the proteins, the DNA depletion of all tested columns increased with a higher cut‐off. Consequently, the lowest DNA amounts in the flow‐through were determined with all 5000 kDa cut‐off columns, with the exception of DUO S. The latter was not capable of retaining notable DNA amounts under the applied process conditions, due to the negative charge of the core ligands at a neutral buffer pH.

In summary, the VP A and B columns delivered the highest capacity compared to the columns tested, considering the applicable amount of feed volume and the best impurity removal, at the cost of an impaired product yield. For the other columns, the infective virus recovery was comparable at 70%–90%. The differences in their capacity and in their protein and DNA depletion deviated only slightly (Table 1). An increased pore size of the shell seemed beneficial, as the DUO Q 5000 column performed best with respect to all tested aspects. For virus purifications at a neutral pH, RAM materials with a cation exchange core are less suitable, as especially the separation of contaminating host cell DNA and product represent a significant challenge.

### 3.2 RAM column performance with regeneration and cleaning

Following the evaluation of the general column suitability for the model virus purification, the selected RAM columns were cleaned (NaOH and H_2_O) and re‐equilibrated up to two times for an additional usage. Only the largest available cut‐offs were evaluated for each column, as they performed best in the previous study, and it can be assumed that these show the greatest regeneration potential due to a higher diffusion rate [28].

**FIGURE 2:**
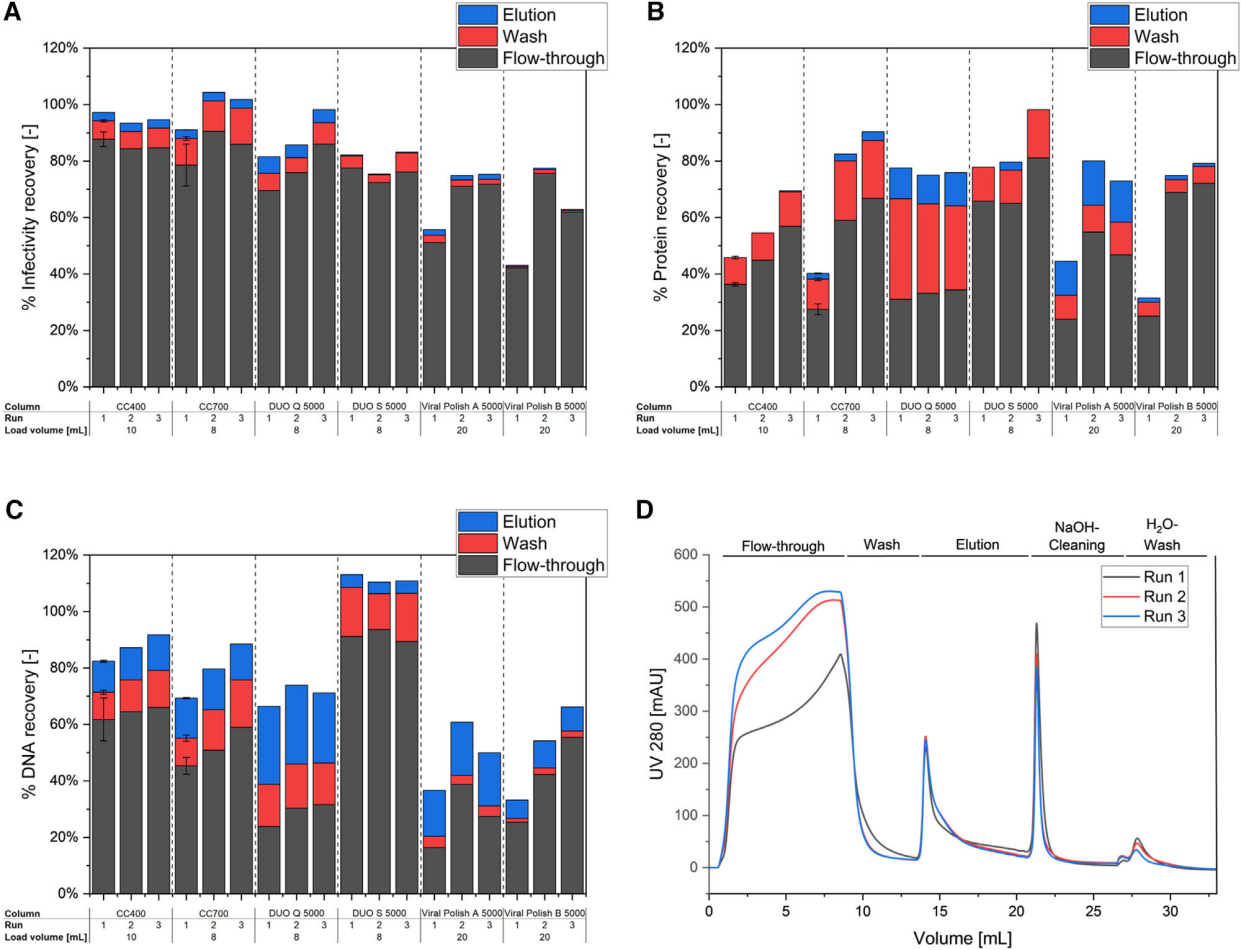
Effect of column regeneration on the purification efficiency for different RAM columns. For each 1 mL column, three subsequent purification runs were done, using clarified Orf virus harvests with an intermediate column cleaning (1 M NaOH followed by a water wash). Accordingly, *Run 2* equals one regeneration cycle, and *Run 3* describes the third use of the same column (two rounds of purification and regeneration). The percentage recovery of (A) infective viruses, (B) total protein, and (C) total DNA content was measured in relation to the feed. All experiments were done once, as a proof of concept, except the first runs of CC400 and 700, which were done in triplicates to estimate deviations due to column batch‐to‐batch variations. An example chromatogram, depicting the changes after cleaning, is given for the CC700 regarding the protein breakthrough based on the UV_280_ signal (D).

The results lined out two major observations: Firstly, the recovery of infective viruses in the product fraction is above 80%, irrespective of repeated loading for all columns, except the VP A and B columns (Figure 2A). For the latter two columns, about 42%–51% of the product were recovered during the first column use, and about 65%–76% during the second and third run. For these columns, it can be assumed that a higher loading volume will also have a positive effect on the virus yield, but at the expense of product purity. Secondly, it was observed that the amount of protein and DNA, retained on the column, is reduced for subsequent runs, leading to a diminished purification (Figure 2B,C). The effect is strongest for the CC700 and the VP A and B columns, with differences in the protein breakthrough from the first to the last run of 40%–50%. The quantification data, obtained from offline analytics, confirmed the online UV_280_ signal, which showed an increased protein breakthrough for equal loading volumes (shown for CC700, Figure 2D). For the DNA recovery, the effect was less distinct, but still accounted for up to 30%. The stated observations of an increasing virus recovery and a reduced purification performance can be attributed to a cumulative irreversible binding of contaminants, either within the core, or due to an unspecific binding on the hydrophilic particle shell. Evidently, this effect cannot be prevented by a high salt elution, or by a cleaning procedure using NaOH. Current literature on the CC700 column, but also the manufacturers’ instructions, indicate an additional implementation of organic solvents during the regeneration, such as (iso‐)propanol [21, 31, 32], however, no results of the column performance after regeneration were presented. Furthermore, in preceding studies, we could not detect a notable benefit of this approach during ORFV purifications (data not shown), and the application of organic solvents extends the column cleaning cycle due to the stepwise adjustments of the aqueous and organic mobile phases. Hence, it was decided to use a less complex regeneration scheme to compare the reusability of the different columns. Depending on the feed composition, the columns might be suitable for multiple re‐use with an adequately optimized cleaning procedure for other viruses. Otherwise, a single‐use application is clearly recommended, as was previously suggested elsewhere for the CC700 [23, 28].

In contrast to these observations, the DUO Q column enabled a constant impurity recovery for all three runs, with 31%–35% of the protein and 21%–28% of the DNA remaining in the flow‐through. Remarkable is the larger quantity of proteins and DNA exiting the column during wash (up to 36% protein, 20% DNA) and elution (up to 13% protein, 30% DNA) (Figure 1, 2, Table 1). This results in an overall reduced burden of molecules attached to the column, which requires a removal during the cleaning procedure. Accordingly, the cleaning can be considered more effective, as also the risk for pore blockage, due to clogging of proteins and DNA, is reduced. A major cause for this effect might be the ligand chemistry of the core. All other tested columns that enabled impurity depletion, that is, except the DUO S, offered multimodal binding, combining anion exchange and hydrophobic interaction (Table 1). In contrast to these, the DUO Q core allowed only anion exchange interactions. Hence, the adsorption can be considered as generally weaker than for the multimodal columns. Additionally, it is possible to elute anion exchangers by an increase of the buffers’ ionic strength [5, 10]. This facilitates a removal of the impurities already during the wash and elution steps, as can be derived from modeling approaches for protein purifications by anion exchangers [33], or as seen in gradient elution steps with an increased NaCl concentration for influenza A viruses [9].

In summary, the various columns showed different regeneration potentials when subjected to a simplified cleaning procedure comprising NaOH and water. Most of the columns had a reduced capacity for total protein and DNA after the cleaning, and are thus preferential for a single‐use application. The DUO Q 5000 column, on the contrary, offered only a minor impairment of the virus recovery and impurity depletion upon the cleaning procedure. Hence, re‐using the DUO Q 5000 column is feasible, although it has to be evaluated how many application cycles are possible for different products, depending on the feed material.

### 3.3 Scale‐up RAM column performance

In the last step, larger scale columns of the CC700 and the MC60 were evaluated regarding their capacity, virus recovery, and impurity depletion. This was done to allow a comparison between the different scales (CC700), and to assess the MC60, which was commercially not available in a 1 mL size. Both columns were loaded until the protein breakthrough increased to about 10%–20% of the maximum, just as for the 1 mL columns. Accordingly, the CC700 could be loaded with about 41 mL (8.7 CV), and the MC60 with about 34 mL (8 CV). Thus, the loading volumes can be considered to be comparable for these two columns, as well as for the two different tested CC700 scales with 8 CV of feed material being loaded on the 1 mL column, and 8.7 CV loaded on the 4.7 mL column.

**FIGURE 3:**
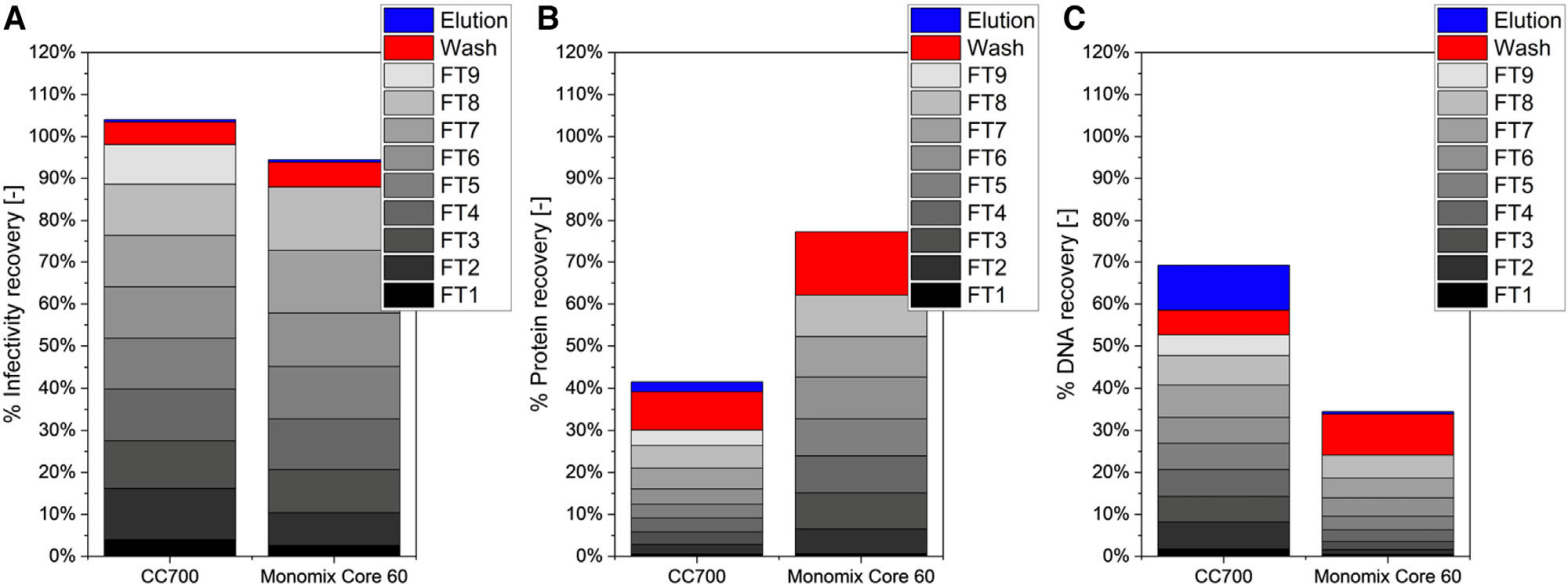
Comparison of larger‐scale RAM column performance. The Monomix Core 60 (4.2 mL column volume) was compared to the CC700 (4.7 mL column volume). Evaluated were the recovery of infective viruses (A), total protein (B), and DNA (C) in the flow‐through (FT), wash, and elution fractions. The recovery is given in relation to the feed. Both runs were performed once, as a proof of concept. The FT was fractionated into samples of 1 column volume. The different shading of the individual FT fractions is for a better visual differentiation.

For both tested columns, CC700 and MC60, about 90% of the infective product viruses were found in the flow‐through (Figure 3A). Concerning the impurity removal, a more differentiated result was determined. The CC700 provided a superior protein removal, as about 30% of the proteins remained in the flow‐through, in comparison to 61% for the MC60 column (Figure 3B). In contrast, the DNA depletion was advanced for the MC60, as only 24% of the initial DNA eluted along with the product, which, however, was twice as high as for the CC700 with 53% (Figure 3C). Notably, on both columns, the level of the impurities in each fraction of the flow‐through increased during the course of loading, indicating a saturation of binding sites, or a limited accessibility of the ligands due to diffusion limitations. The latter could be improved by adjusting the processing flow rate. In our experiments, we aimed to work with flow rates of 1 CV per mL to allow comparisons based on a simplified process setup. However, for the CC700 the volumetric adjustment of the flow‐rate during the scale‐up exceeded the operational range of the column, which is why not 1 CV min^−1^ was used, but the maximum possible flow rate. This was done because a high throughput of feed material was desired. In order to further optimize the impurity retention and to avoid diffusion limitations, the flow rate could be further decreased. A comprehensive optimization, however, was not part of the column screening presented here.

Finally, it was determined for both columns that the retained impurities could only be removed from the column to a maximum of 20% during wash and elution. Particularly for the MC60, the elution did not enable a notable impurity recovery (Figure 3). With the results from the previous section, it can be assumed that a rigorous optimization of the regeneration procedure would be required to enable a repeated usage. During most pharmaceutical production processes for viruses, the main issue for product purity is the residual DNA [7]. Accordingly, the MC60 would be a better choice for final polishing, rather than being used during primary purification as done in this study.

For the CC700, the data suggests a high comparability for different scales, as not only the capacity of the column (see above), but also the ORFV recovery and the impurity depletion were similar for both column sizes. In addition to the technical triplicate runs on a small scale, this indicates a high reproducibility of the CC700 application. As the separation principle is the same for all RAM techniques with no direct binding interaction between the virus and the resin due to the inert shell material, a straightforward scalability can be assumed for the other tested materials as well. However, this assumption has to be verified in upcoming evaluations. Previously, the CC700 was applied for various viral targets and different column sizes [15, 18, 21, 23, 31, 34]. However, to the best of our knowledge, no direct comparison of purification scales has been described for the same product virus. Although it will be necessary to analyze additional scales, the present data suggests an easy translation to different production scales, if the loaded feed amount and the residence time during loading are adjusted with regard to the resin bed volume.

## 4 CONCLUDING REMARKS

RAM greatly improve DSP procedures for virus‐based therapeutics and enable a fast and gentle processing of the product while removing major contaminants, such as DNA and proteins. We evaluated the performance of various commercially available RAM columns for the purification of a clarified ORFV harvest with regard to the impurity removal and product recovery. The tested RAM columns feature individual characteristics in terms of pore size distribution in the shell and ligand chemistry within the cores. This allows a tailored process integration of the RAM technology depending on the product requirements, as DNA and proteins are depleted with different efficiencies. For the model virus, ORFV, RAM materials with anion exchange or multimodal ligand interactions are preferable, as cation exchange core ligands do not enable the retention of impurities (e.g., host cell DNA) at a neutral pH value, and pH adjustments would affect the stability of the virus. Additionally, in the described studies, a clarified feed solution was applied, which challenges the column with excessive amounts of protein and DNA. The results show that some of the columns might be better suitable for a residual impurity removal (e.g., MC60 for DNA). Furthermore, the data suggests that most of the columns are more suitable for a single‐use application instead of multiple usages, in order to enable a reproducible process conduction. This is caused by an irreversible adsorption of components to the core ligands, which requires an extensive cleaning procedure. For a repeated usage, RAM columns without a multimodal ligand, but with anion exchange binding mechanisms, are preferential, as a streamlined cleaning procedure is possible. For the evaluated model ORFV, CC700 provides the most reliable technique for product recovery and impurity depletion, when applied during a primary purification in single‐use. For repeated usages, the DUO Q 5000 is preferential due to an easier column regeneration. Additionally, the DUO Q 5000 and the MC60 provided a remarkable DNA depletion and would, thus, be particularly suitable at later DSP stages.

In summary, when choosing a suitable column and proper process conditions, for example, considering the residence times, the RAM technology offers a potential platform methodology as part of a DSP procedure for a gentle purification of cell culture‐derived viruses. The results for product yield and contaminant depletion permit the assumption that the technique might take its firm place in viral production processes in the future. The presently commercially available matrices with different characteristics support this possibility.

## CONFLICT OF INTEREST STATEMENT

The authors declare that they have no known competing financial interests or personal relationships that could have appeared to influence the work reported in this paper.

## Data Availability

The data that supports the findings of this study, is available from the authors upon reasonable request.
